# Psychological interventions and mental health outcomes among caregivers of children with intellectual disability: a systematic review and meta-analysis

**DOI:** 10.3389/fpsyg.2026.1883919

**Published:** 2026-08-18

**Authors:** Udeme Samuel Jacob, Mbulaheni Obert Maguvhe, Jace Pillay, Adewuyi Habeeb Omoponle

**Affiliations:** 1 Department of Inclusive Education, School of Educational Studies, College of Education, University of South Africa, Pretoria, South Africa; 2 Faculty of Education, University of Johannesburg, Johannesburg, South Africa; 3 Department of Educational Psychology, Faculty of Education, University of South Africa, Pretoria, South Africa

**Keywords:** caregivers, cognitive behavioral therapy, intellectual disability, mental health, mindfulness intervention, parenting stress

## Abstract

**Introduction:**

Mothers and caregivers of children with intellectual and developmental disabilities often experience depression, anxiety, parenting stress, emotional exhaustion, and reduced psychological wellbeing because of prolonged caregiving demands, behavioral difficulties, financial strain, and social stigma. Psychological and behavioral interventions have increasingly been introduced to improve caregiver mental health outcomes. This study examined the effectiveness of these interventions on the mental health of mothers and caregivers of children with intellectual and developmental disabilities.

**Methodology:**

The study adopted a systematic review and meta-analysis design guided by PRISMA procedures. Electronic databases, including PubMed, PsycINFO, Scopus, Web of Science, CINAHL, Embase, ERIC, and Google Scholar, were searched for relevant studies published up to December 2025. Fourteen studies involving more than 740 participants met the eligibility criteria. Data extraction, quality appraisal, subgroup analyses, sensitivity analyses, and publication bias assessments were conducted using standard meta-analytic procedures.

**Results:**

The findings demonstrated that psychological and behavioral interventions significantly improved depression, anxiety, parenting stress, mental health, and wellbeing outcomes among caregivers. Cognitive Behavioral Therapy produced the strongest reductions in depressive symptoms and irrational beliefs, while mindfulness and psychoeducational interventions improved stress management, emotional regulation, and parenting self-efficacy.

**Discussion:**

The findings suggest that caregiver-focused psychological interventions may strengthen emotional resilience, coping capacities, and family wellbeing. The review highlights the importance of integrating evidence-based mental health interventions into disability support and family-centered rehabilitation services.

## 1 Introduction

Intellectual disability (ID) is a neurodevelopmental condition characterized by significant limitations in intellectual functioning and adaptive behavior that emerge during the developmental period (Green and Losh, 2025; Courtenay et al., 2024). The condition affects conceptual, practical, and social functioning, thereby influencing independent participation in daily activities and community life (Imran and Aamer, 2025; Lee et al., 2020). Intellectual disability remains a major public health and educational concern affecting approximately 1–3% of the global population, especially among children and adolescents (Anderson and Grossman, 2011; Tuffrey-Wijne, 2025). Genetic abnormalities, neurological conditions, prenatal complications, infections, and environmental influences have all been associated with the development of intellectual disability (Gopalan, 2021; Tarani et al., 2022).

Children with intellectual disability frequently experience deficits in communication, reasoning, adaptive functioning, social interaction, and self-care skills, often requiring continuous supervision and support from caregivers (Korb et al., 2022; Carneiro et al., 2023). Many children with ID additionally present with behavioral, emotional, and psychiatric difficulties that further increase caregiving demands and family stress (Hashim et al., 2016; Broberg, 2011). Consequently, caregiving responsibilities commonly involve long-term engagement with healthcare services, rehabilitation programs, educational systems, and social support structures, thereby placing substantial emotional and financial strain on families (Tomaz et al., 2017; Lu et al., 2026).

Within many families, mothers assume the primary caregiving role for children with intellectual disability and are responsible for behavioral management, educational support, healthcare coordination, and daily supervision (Raliphaswa et al., 2022; Sari and Basbakkal, 2009). The persistent nature of caregiving responsibilities may negatively affect maternal physical health, emotional stability, occupational functioning, and social participation (Dindar et al., 2016; Syed et al., 2020). Mothers frequently report feelings of guilt, hopelessness, uncertainty regarding the future, and fear relating to the long-term wellbeing of their children (Gallagher et al., 2008; Inan Budak et al., 2018). Financial burdens associated with healthcare services, specialized education, and transportation may further intensify caregiver stress, particularly in low-resource settings where access to disability-related services remains limited (Marais et al., 2022; Tomaz et al., 2017).

The social experiences of mothers caring for children with intellectual disability are additionally shaped by stigma, discrimination, and negative societal attitudes toward disability (Broberg, 2011; Ardani et al., 2022). Misconceptions regarding intellectual disability may contribute to social exclusion and inadequate emotional support for families. Mothers may withdraw from social interactions because of embarrassment, fear of judgment, or negative community reactions (Raliphaswa et al., 2022; Miezah et al., 2026). Reduced social connectedness and inadequate support networks may therefore increase emotional vulnerability and psychological distress among caregivers (Abredari et al., 2026; Barratt et al., 2025).

Research evidence increasingly demonstrates that caregiving for children with intellectual disability is associated with elevated levels of depression, anxiety, stress, emotional exhaustion, and reduced quality of life among mothers (Rydzewska et al., 2021; Mohammadi et al., 2023). Maternal depressive symptoms are often associated with chronic caregiving stress, disrupted family functioning, behavioral challenges, and uncertainty surrounding the child’s long-term independence (Papadopoulos, 2024; Adams et al., 2018). Persistent depression may reduce emotional availability, parenting confidence, and family cohesion, thereby negatively affecting both caregiver wellbeing and child developmental outcomes (Kim et al., 2003; Gallagher et al., 2008). Anxiety similarly represents a major psychological concern among caregivers and may arise from concerns relating to behavioral management, educational access, financial instability, and future caregiving responsibilities (Ardani et al., 2022; Miezah et al., 2026).

Parenting stress and emotional exhaustion are also highly prevalent among caregivers of children with intellectual disability (Barratt et al., 2025; Syed et al., 2020). Daily caregiving responsibilities frequently involve managing behavioral difficulties, assisting with self-care activities, attending therapy appointments, and navigating healthcare and educational systems (Lu et al., 2026; Dindar et al., 2016). The continuous nature of these responsibilities may contribute to burnout, emotional depletion, fatigue, and feelings of helplessness among caregivers (Abredari et al., 2026; Barratt et al., 2025). Mothers experiencing high caregiver burden commonly report poorer mental health outcomes, lower life satisfaction, and reduced participation in social and recreational activities (Sari and Basbakkal, 2009; Syed et al., 2020).

The growing prevalence of psychological distress among caregivers highlights the urgent need for evidence-based mental health interventions and family support services (Kumari et al., 2025; Yavuz and Sümer Dodur, 2026). Psychological support programs may assist caregivers in developing adaptive coping skills, emotional resilience, stress management strategies, and positive parenting practices (Hastings and Beck, 2004; Lobato et al., 2025). Access to counseling, peer support networks, and family-centered services may also reduce emotional isolation and strengthen caregiver wellbeing (Doyle, 2022; Raliphaswa, 2026).

Therapeutic interventions targeting caregiver mental health have demonstrated promising outcomes in reducing stress, anxiety, depression, and caregiver burden among parents of children with developmental disabilities (Chua and Shorey, 2022; Yang et al., 2025). Cognitive Behavioral Therapy (CBT), mindfulness-based interventions, Acceptance and Commitment Therapy, psychoeducation, and behavioral parent training programs have all been associated with improved emotional regulation and psychological wellbeing among caregivers (Neece and Lima, 2016; Gyereh and Shukla, 2024). Group-based interventions additionally provide opportunities for emotional sharing, social connectedness, and mutual support among caregivers experiencing similar challenges (Hastings and Beck, 2004; Osborn et al., 2025).

Family-centered mental health approaches recognize the interconnected nature of family wellbeing and emphasize collaborative partnerships between healthcare professionals and families (Mestre et al., 2024; Dempsey et al., 2009). These approaches focus on empowering caregivers, improving communication, strengthening coping resources, and promoting positive family functioning (Mestre et al., 2025a; Mestre et al., 2025b). Family-centered interventions may additionally improve parent-child relationships and facilitate better developmental outcomes for children with intellectual disability (Lo et al., 2023; Sutherland et al., 2025).

Several psychological and behavioral interventions have been developed to address the mental health difficulties experienced by caregivers of children with intellectual disability. Cognitive Behavioral Therapy remains among the most widely researched interventions and focuses on modifying maladaptive thoughts, beliefs, and behavioral patterns associated with depression, anxiety, and stress (Corcoran and Walsh, 2009; Hopkinson et al., 2019). Mindfulness-based interventions promote emotional acceptance, stress reduction, and present-moment awareness through mindfulness practices and meditation techniques (Apolinário et al., 2020; Osborn et al., 2025). Psychoeducation programs provide caregivers with information regarding disability management, behavioral support strategies, emotional regulation, and available support services (Totsika et al., 2025; Neece and Lima, 2016). Counseling, social support interventions, behavioral parent training, and stress management programs have additionally been utilized to improve family functioning and reduce caregiver burden (Gyereh and Shukla, 2024; Adams and Jahoda, 2019).

Recent developments in digital mental health have increased the use of online interventions, mentored self-help programs, and technology-assisted therapies targeting caregiver wellbeing (Amer et al., 2024; Apolinário et al., 2020). These approaches may improve accessibility to psychological services for caregivers experiencing transportation, financial, or time-related barriers. Despite the growing body of intervention research, findings across studies remain inconsistent, thereby necessitating systematic synthesis of available evidence regarding the effectiveness of psychological and behavioral interventions for mothers and caregivers of children with intellectual and developmental disabilities.

This study is anchored on Stress and Coping Theory, Family Systems Theory, and Cognitive Behavioral Theory. Stress and Coping Theory explains how individuals appraise stressful situations and utilize coping strategies to manage emotional distress (Folkman, 2012; Matthieu and Ivanoff, 2006). Family Systems Theory views the family as an interconnected emotional unit in which changes affecting one member influence the functioning of the entire household (Watson, 2012; Pfeiffer and In-Albon, 2022). Cognitive Behavioral Theory additionally explains that dysfunctional thoughts and maladaptive behavioral patterns contribute substantially to emotional distress and psychological difficulties (Early and Grady, 2017; Mandel et al., 2024). Collectively, these theories provide a framework for understanding the psychological experiences of caregivers and the mechanisms through which interventions may improve mental health outcomes.

### 1.1 Objectives of the study

The main objective of this systematic review and meta-analysis is to examine the effectiveness of psychological and behavioral interventions on the mental health outcomes of mothers and caregivers of children with intellectual and developmental disabilities. Specifically, the study seeks to:

1. examine the characteristics of studies investigating psychological and behavioral interventions for mothers and caregivers of children with intellectual and developmental disabilities;
2. identify the intervention approaches, delivery formats, duration, and therapeutic components utilized across the included studies;
3. identify the mental health outcomes and assessment instruments used across the studies;
4. evaluate the effectiveness of psychological and behavioral interventions on caregiver mental health outcomes; and
5. examine subgroup effects, sensitivity analyses, risk of bias, and publication bias across the included studies.

## 2 Methodology

### 2.1 Research design

This study adopted a systematic review and meta-analysis design to synthesize empirical evidence on interventions targeting the mental health of parents, mothers, and caregivers of children with intellectual disability and related developmental disabilities. The systematic review approach enabled the identification, appraisal, and synthesis of relevant studies using transparent and reproducible procedures, while the meta-analysis statistically pooled findings from eligible studies to estimate the overall effectiveness of interventions on mental health outcomes. The review was conducted in accordance with the Preferred Reporting Items for Systematic Reviews and Meta-Analyses (PRISMA) guidelines to ensure methodological transparency and rigor throughout the review process (Ibrahim et al., 2012; Ghafari et al., 2016). Although the present review was not prospectively registered in PROSPERO or the Open Science Framework (OSF), all methodological procedures, including the objectives, eligibility criteria, search strategy, data extraction procedures, quality appraisal methods, and statistical analysis plans, were predefined before the commencement of the review in order to minimize bias and ensure methodological consistency.

### 2.2 Eligibility criteria

Eligibility criteria were established using the Population, Intervention, Comparison, Outcomes, and Study Design (PICOS) framework to guide the inclusion and exclusion of studies. Studies were included if they involved parents, mothers, fathers, or caregivers of children diagnosed with intellectual disability or developmental disability. Eligible studies included experimental, quasi-experimental, pilot intervention, and randomized controlled trials that reported mental health outcomes such as depression, anxiety, stress, psychological wellbeing, emotional distress, self-compassion, or quality of life. Peer-reviewed journal articles and eligible gray literature with sufficient methodological information were considered for inclusion. Only studies published in English language and those reporting adequate statistical information necessary for effect size calculation were included in the review.

Studies were excluded if they lacked intervention components or employed qualitative, narrative review, systematic review, or meta-analytic designs. Case reports, editorials, conference abstracts, opinion papers, and studies with inaccessible full texts were also excluded. In addition, studies involving interventions targeted exclusively at children without caregiver participation were not considered eligible for inclusion in the review. The exclusion of dissertations and inaccessible reports was supported by previous systematic review evidence indicating that such exclusions may not substantially influence review outcomes (Jacob et al., 2022b; Jacob et al., 2022a; Jacob et al., 2023; Jacob-Udeme et al., 2025; Vickers and Smith, 2000).

### 2.3 Information sources and electronic databases

A comprehensive literature search was conducted across multiple electronic databases to identify relevant studies published from database inception to December 2025. The databases searched included PubMed, MEDLINE, PsycINFO, Scopus, Web of Science, CINAHL, ERIC, Embase, Cochrane Library, ScienceDirect, SpringerLink, Taylor and Francis Online, Wiley Online Library, JSTOR, ProQuest Dissertations and Theses, Google Scholar, African Journals Online, Dimensions, Semantic Scholar, and OpenGrey. The inclusion of multiple databases ensured broad coverage of medical, psychological, educational, rehabilitation, and social science literature related to intellectual disability and caregiver mental health interventions.

To improve the comprehensiveness of the review, manual searching procedures were also employed. Reference lists of eligible studies and previous reviews were examined to identify additional relevant studies. Citation tracking was conducted using Google Scholar and Web of Science to identify newer studies citing key articles. Hand-searching of leading journals in special education, rehabilitation, developmental disabilities, psychology, and mental health was additionally performed to minimize the possibility of omitting relevant studies.

### 2.4 Search strategy

The search strategy combined Medical Subject Headings (MeSH), controlled vocabulary terms, keywords, and Boolean operators to maximize the sensitivity and specificity of the search process. Major keywords used in the search included “mental health,” “mothers,” “caregivers,” “intellectual disability,” “developmental disability,” “intervention,” “treatment,” “therapy,” “psychological wellbeing,” “depression,” “anxiety,” and “stress.” Additional synonymous and truncated terms were incorporated where necessary to increase retrieval efficiency across databases.

Boolean operators were utilized systematically during the search process. The operator AND was used to combine major concepts, OR was used to include synonymous or related terms, while NOT was employed to eliminate irrelevant studies from the search results. An example of the search string used across databases was: (“mothers” OR “caregivers” OR “parents”) AND (“intellectual disability” OR “developmental disability”) AND (“mental health” OR depression OR anxiety OR stress OR wellbeing) AND (treatment OR intervention OR therapy). The complete database-specific search strategies are presented in Supplementary Table 1. Minor modifications were made across databases to accommodate differences in indexing systems and database-specific search requirements.

### 2.5 Study selection process

All retrieved records were imported into EndNote reference management software for organization and duplicate removal. After duplicates were removed, the remaining studies underwent title and abstract screening based on the predefined eligibility criteria. Potentially relevant studies were screened for full text.

**FIGURE 1:**
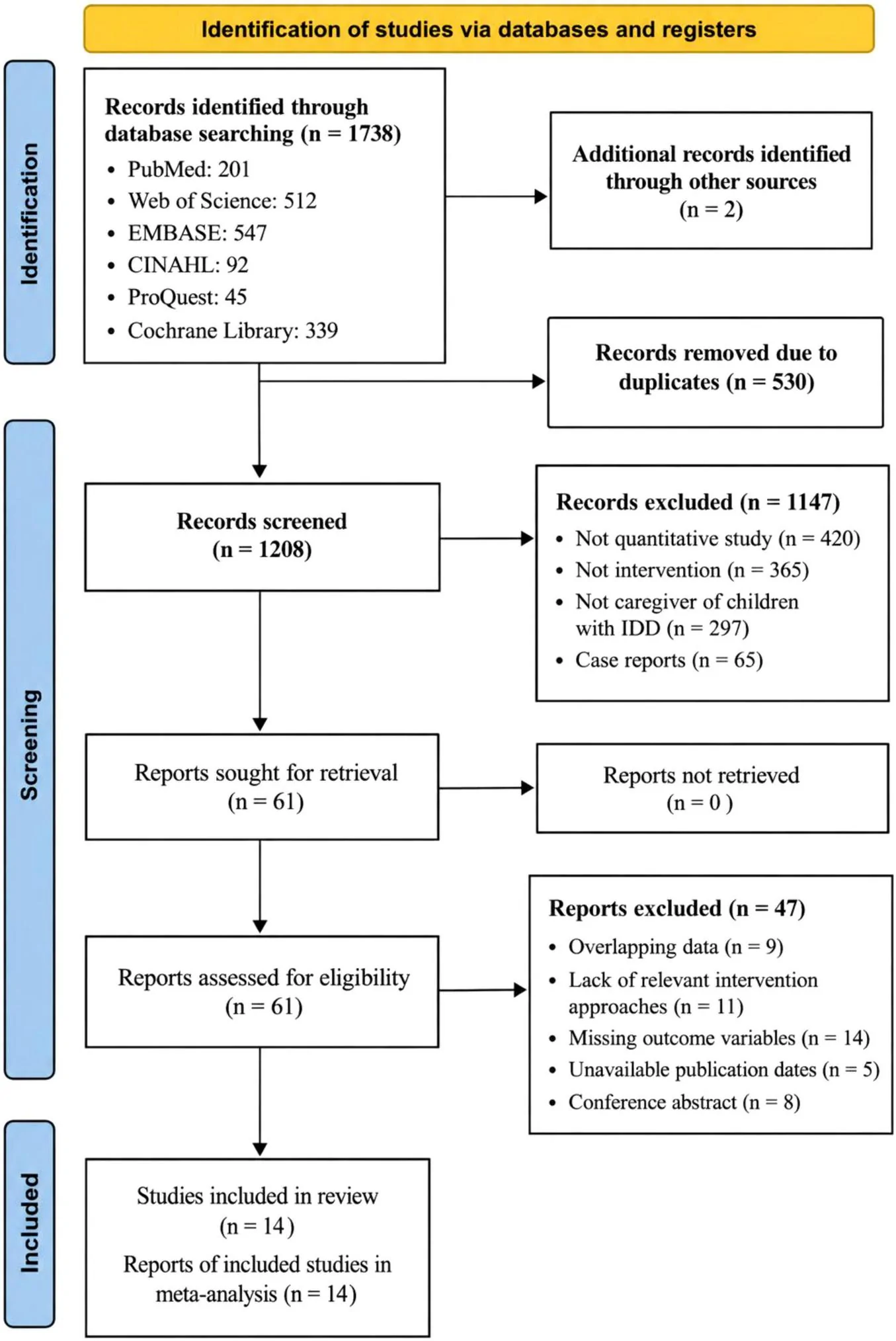
PRISMA flow diagram of study selection.

Two independent reviewers conducted all stages of screening to minimize selection bias. Disagreements between reviewers were resolved through discussion and consensus. Where disagreements persisted, a third reviewer was consulted. The study selection procedures were guided by established systematic review recommendations for evidence synthesis and study eligibility assessment (Vickers and Smith, 2000). The entire selection process was documented using the PRISMA flow diagram (see Figure 1).

A total of 1,738 records were identified through database searching, including PubMed (201), Web of Science (512), EMBASE (547), CINAHL (92), ProQuest (45), and Cochrane Library (339). Two additional records identified through other sources were found, bringing the total to 1,740. After removing 530 duplicates, 1,210 records remained for screening. Following title and abstract screening, 1,149 studies were excluded because they were not quantitative studies, lacked intervention components, did not involve caregivers of children with intellectual disability, or were case reports. Sixty-one reports were sought for retrieval, and all were successfully retrieved and assessed for eligibility. During full-text assessment, 47 reports were excluded due to overlapping data, absence of mindfulness-based interventions, failure to report relevant outcome variables, unavailable publication dates, or conference abstract status. Fourteen studies met the eligibility criteria and were included in the systematic review and meta-analysis.

### 2.6 Data extraction

The researchers developed a structured data extraction form to ensure consistency in data collection. Two reviewers independently extracted data from the included studies. Extracted information included author and publication year, country of study, study design, sample size, participant characteristics, type of intervention, duration of treatment, outcome measures, follow-up period, main findings, and effect sizes. Where data were incomplete or unclear, attempts were made to contact corresponding authors for clarification. Any discrepancies during extraction were resolved through discussion between reviewers.

### 2.7 Outcome measures

The review focused on caregiver mental health outcomes measured using standardized instruments. The major outcome measures included depression scales such as the Beck Depression Inventory and the Patient Health Questionnaire, anxiety scales such as the State-Trait Anxiety Inventory and the Generalized Anxiety Disorder Scale, parenting stress measures such as the Parenting Stress Index, psychological wellbeing scales, self-compassion scales, and quality-of-life measures. Only studies employing validated and reliable instruments were included in the review.

### 2.8 Quality appraisal

Methodological quality assessment was conducted independently by two reviewers using three major appraisal tools. The Joanna Briggs Institute (JBI) critical appraisal checklist was used to evaluate methodological rigor in quasi-experimental studies (Aromataris and Munn, 2020). The Cochrane Risk of Bias Tool was used to assess selection bias, performance bias, detection bias, attrition bias, and reporting bias in randomized controlled trials (Higgins et al., 2022). The Physiotherapy Evidence Database (PEDro) Scale was additionally applied to assess internal validity and statistical reporting quality (Maher et al., 2003). Studies were categorized into high, moderate, and low quality based on appraisal scores. Disagreements in quality ratings were resolved through discussion and consensus between the reviewers.

### 2.9 Data synthesis and statistical analysis

Narrative synthesis was used to summarize study characteristics, intervention approaches, outcome measures, and major findings. The synthesis highlighted similarities and differences among interventions, participant characteristics, and study outcomes. Meta-analysis was conducted using Review Manager (RevMan) and Comprehensive Meta-Analysis software. Hedges’ g and Standardized Mean Difference (SMD) were computed as effect size indicators due to variations in measurement scales across studies. A random-effects model was adopted because substantial clinical and methodological heterogeneity was anticipated among included studies.

Statistical heterogeneity was assessed using Cochran’s Q statistic and the I^2^ statistic. I^2^ values of 25, 50, and 75% represented low, moderate, and high heterogeneity, respectively. Significant heterogeneity prompted further subgroup analyses. Publication bias was assessed through visual inspection of funnel plots and Egger’s regression test. Funnel plot asymmetry was interpreted cautiously, as it may also arise from heterogeneity and small-study effects.

Subgroup analyses were conducted to examine potential moderators of intervention effectiveness, including treatment type, intervention duration, severity of the child’s disability, country income level, and online versus face-to-face delivery. Sensitivity analyses were conducted using leave-one-out procedures and influence diagnostics to determine the robustness and stability of pooled effect estimates. Studies with extremely large effect sizes or high risk of bias were sequentially removed to evaluate their influence on the overall findings.

## 3 Results

### 3.1 Characteristics of included studies

Table 1 presents the characteristics of the studies included in the systematic review and meta-analysis. The studies were conducted across several countries including the United States (Chronis et al., 2006; Raulston et al., 2019; Bazzano et al., 2013; Magaña et al., 2015), China (Zhou et al., 2018), Australia (Tonge et al., 2006; Wong et al., 2011), Saudi Arabia (Hemdi and Daley, 2017), Hong Kong (Lo et al., 2017; Lo et al., 2024), the United Kingdom (Lovell et al., 2016), and Iran (Dehghani, 2016; Poorseyed et al., 2017; Mokhtaree et al., 2016). The geographical distribution reflects increasing international attention toward interventions aimed at improving the mental health of mothers and caregivers of children with intellectual and developmental disabilities. Most studies adopted randomized controlled trial designs (Chronis et al., 2006; Tonge et al., 2006; Hemdi and Daley, 2017; Lo et al., 2017), while pilot intervention and quasi-experimental studies were also represented (Raulston et al., 2019; Dehghani, 2016; Zhou et al., 2018).

**TABLE 1:** Characteristics of included studies.

Author	Country	Participants	Mean age/range	Child diagnosis	Design	Intervention	Duration	Outcomes
Chronis et al. (2006)	United States	Mothers of children with ADHD	Not clearly stated	ADHD	Randomized controlled trial	Coping with depression course (CBT-based)	Conducted alongside the summer treatment program	Maternal depressive symptoms, self-esteem, child-related cognitions, and family impairment
Zhou et al. (2018)	China	64 parents of ASD children and 63 parents of typically developing children	Not clearly stated	Autism spectrum disorder	Pilot intervention study	Family-focused psychoeducational therapy (FFPT)	4 Weeks	Parenting self-efficacy, anxiety, depression
Raulston et al. (2019)	United States	3 Mother-child dyads	Not clearly stated	Autism spectrum disorder	Single-case randomized multiple baseline design	Mindfulness-infused behavioral parent training	Embedded within natural family routines	Parent stress, behavioral strategy use, and child challenging behavior
Tonge et al. (2006)	Australia	Parents of preschool children with autism (*n* = 105)	Parents of children aged 2–5 years	Autism	Randomized controlled trial	Parent education and behavior management intervention	20 Weeks	Mental health, anxiety, insomnia, somatic symptoms, family dysfunction
Hemdi and Daley (2017)	Saudi Arabia	62 mothers of children with ASD	23–52 Years	Autism spectrum disorder	Multisite randomized controlled trial	Psychoeducation intervention delivered via WhatsApp	One face-to-face session and four virtual sessions	Parenting stress, depression, anxiety, happiness
Wong et al. (2011)	Australia	39 Chinese parents of children with intellectual disabilities	Not clearly stated	Intellectual disability	Follow-up intervention study	Cognitive-behavioral treatment groups	Follow-up at 6 months	Parenting stress, mental health, quality of life, dysfunctional attitudes
Lo et al. (2024)	Hong Kong	43 Parents of children with ADHD	Not clearly stated	ADHD	Pilot mixed-methods randomized study	Online mindfulness-based program	28 Days	Child ADHD symptoms, emotion regulation, parental wellbeing
Bazzano et al. (2013)	United States	76 Parents/caregivers	Not clearly stated	Developmental disabilities	Community-based intervention study	Mindfulness-based stress reduction (MBSR)	8 Weeks	Perceived stress, parental stress, mindfulness, wellbeing
Lo et al. (2017)	Hong Kong	180 Parents of preschool children with developmental disabilities	Not clearly stated	Developmental Disabilities	Randomized controlled trial	Brief mindfulness-based program	6 Weekly sessions	Stress, depression, dysfunctional parent-child interaction
Magaña et al. (2015)	United States	Latina mothers of youths/adults with IDD	Not clearly stated	Intellectual and developmental disabilities	Randomized controlled design	Culturally sensitive health education intervention	3-Month post-test	Health self-efficacy, health behaviors, stress, depressive symptoms
Lovell et al. (2016)	United Kingdom	37 Caregivers of children with autism	Not clearly stated	Autism	Pilot intervention study	Written benefit-finding intervention	Three writing sessions	Anxiety, depression, and psychological wellbeing
Dehghani (2016)	Iran	30 Mothers of autistic children	Not clearly stated	Autism spectrum disorder	Quasi-experimental design	Cognitive Behavioral Therapy	10 Weekly 75-min sessions	Mental health, irrational beliefs
Poorseyed et al. (2017)	Iran	40 Mothers of children with severe intellectual disability	Not clearly stated	Severe intellectual disability	Quasi-experimental design	Group cognitive-behavioral training	Not clearly stated	Psychological problems, irrational beliefs
Mokhtaree et al. (2016)	Iran	60 Parents of children with intellectual disabilities	Not clearly stated	Intellectual disability	Experimental design	Stress management training with CBT style	Six sessions over 3 weeks	Mental health, stress, depressive symptoms

The included studies involved more than 740 participants comprising mothers, caregivers, and parents of children diagnosed with autism spectrum disorder, intellectual disability, developmental disabilities, and attention-deficit/hyperactivity disorder. Sample sizes varied considerably, ranging from 3 mother-child dyads in Raulston et al. (2019) to 180 parents in Lo et al. (2017). Some studies focused specifically on mothers of children with autism spectrum disorder (Hemdi and Daley, 2017; Dehghani, 2016), whereas others involved broader caregiver populations (Bazzano et al., 2013; Wong et al., 2011).

Different intervention approaches were identified across the studies. Cognitive Behavioral Therapy-based interventions demonstrated improvements in depressive symptoms, irrational beliefs, parenting stress, and family functioning (Chronis et al., 2006; Dehghani, 2016; Poorseyed et al., 2017). Mindfulness-based interventions showed positive effects on parental stress, emotional regulation, and wellbeing (Raulston et al., 2019; Bazzano et al., 2013; Lo et al., 2017; Lo et al., 2024), while psychoeducational interventions improved self-efficacy, anxiety, depression, and happiness (Zhou et al., 2018; Hemdi and Daley, 2017). Intervention duration ranged from 3 to 20 weeks, with delivery modes including face-to-face, online, hybrid, and home-based approaches. Overall, the findings suggest that psychological and behavioral interventions may positively improve the mental health and coping capacities of mothers and caregivers of children with intellectual and developmental disabilities.

### 3.2 Characteristics of interventions

Table 2 presents the intervention approaches implemented across the included studies targeting the mental health of mothers and caregivers of children with intellectual and developmental disabilities. The interventions varied in structure, duration, delivery format, and therapeutic orientation. Cognitive Behavioral Therapy (CBT)-based interventions were frequently reported and focused on modifying maladaptive cognitions, strengthening coping skills, and improving emotional functioning among caregivers (Chronis et al., 2006; Wong et al., 2011; Dehghani, 2016; Poorseyed et al., 2017). Major therapeutic components included cognitive restructuring, coping skills training, depression management, and irrational belief modification (Chronis et al., 2006; Mokhtaree et al., 2016).

**TABLE 2:** Characteristics of interventions.

Study	Treatment type	Sessions	Delivery mode	Components	Follow-up
Chronis et al. (2006)	CBT-based depression intervention	Group sessions	Face-to-face	Cognitive restructuring, coping skills, depression management	5 months
Zhou et al. (2018)	Psychoeducation	Weekly sessions	Face-to-face	Parenting education, emotional support	1 month
Raulston et al. (2019)	Mindfulness-infused parent training	Individualized sessions	Natural family routines	Mindfulness, behavioral parent training	Not clearly stated
Tonge et al. (2006)	Parent education and behavior management	Manualized sessions	Face-to-face	Parent skills training, counseling	6 months
Hemdi and Daley (2017)	WhatsApp psychoeducation	5 Sessions	Hybrid (face-to-face and online)	Psychoeducation, emotional support	8 weeks
Wong et al. (2011)	Group CBT	Group sessions	Face-to-face	CBT techniques, cognitive restructuring	6 months
Lo et al. (2024)	Online mindfulness program	20 Videos and 4 meetings	Online	Psychoeducation, mindfulness exercises	Not clearly stated
Bazzano et al. (2013)	Mindfulness-based stress reduction	Weekly sessions	Community-based	Meditation, stress discussion, stretching	2 months
Lo et al. (2017)	Brief mindfulness program	6 Sessions	Face-to-face	Mindfulness exercises, emotional coping	Not clearly stated
Magaña et al. (2015).	Health education intervention	Educational sessions	Face-to-face	Health promotion, self-care, stress reduction	3 months
Lovell et al. (2016)	Written benefit-finding	3 Writing sessions	Home-based	Emotional disclosure and reflective writing	3 Months
Dehghani (2016)	Cognitive behavioral therapy	10 Sessions	Face-to-face	CBT and irrational belief modification	Post-test only
Poorseyed et al. (2017)	Group CBT training	Group sessions	Face-to-face	CBT and irrational belief reduction	Post-test only
Mokhtaree et al. (2016)	Stress management training	6 Sessions	Face-to-face	Stress management, coping skills	1 month

Mindfulness-oriented interventions were also widely implemented. Raulston et al. (2019) integrated mindfulness practices into behavioral parent training, while Bazzano et al. (2013) employed Mindfulness-Based Stress Reduction involving meditation, stretching exercises, and stress-related discussions. Lo et al. (2017) and Lo et al. (2024) similarly implemented brief and online mindfulness programs incorporating psychoeducation, mindfulness exercises, and emotional coping strategies. These interventions primarily aim to improve emotional regulation, reduce parenting stress, and strengthen psychological wellbeing among caregivers.

Psychoeducational interventions constituted another major treatment category. Zhou et al. (2018) implemented weekly psychoeducational sessions centered on parenting education and emotional support, whereas Hemdi and Daley (2017) adopted a hybrid WhatsApp-based psychoeducational model combining face-to-face and online sessions. Tonge et al. (2006) similarly implemented parent education and behavior management programs involving counseling and parent skills training.

Variation was observed in the delivery modes of the intervention. Face-to-face delivery was predominant across several studies (Chronis et al., 2006; Wong et al., 2011; Dehghani, 2016), although online, hybrid, home-based, and routine-embedded approaches were also identified (Hemdi and Daley, 2017; Lo et al., 2024; Raulston et al., 2019). Intervention duration ranged from three structured writing sessions (Lovell et al., 2016) to programs extending several weeks with follow-up assessments lasting up to 6 months (Tonge et al., 2006; Wong et al., 2011). The interventions addressed psychological outcomes, including stress reduction, emotional wellbeing, adaptive coping, and behavioral management among caregivers of children with intellectual and developmental disabilities.

### 3.3 Outcome measures and assessment instruments

Table 3 presents the outcome measures and assessment instruments used across the included studies. Several validated psychological and behavioral instruments were employed to assess maternal mental health outcomes among mothers and caregivers of children with intellectual and developmental disabilities. Depression was one of the most frequently assessed outcomes and was measured using instruments such as the General Health Questionnaire-28 (GHQ-28), Self-Rating Depression Scale, and Hospital Anxiety and Depression Scale (HADS) (Wong et al., 2011; Dehghani, 2016; Hemdi and Daley, 2017; Lo et al., 2017).

**TABLE 3:** Outcome measures used across studies.

Outcome	Instrument	Reliability	Studies using it
Depression	GHQ-28, Self-rating depression scale, hospital anxiety and depression scale	Acceptable reliability reported	Dehghani (2016); Hemdi and Daley (2017); Lo et al. (2017)
Anxiety	Self-rating anxiety scale, HADS	Acceptable reliability reported	Zhou et al. (2018); Hemdi and Daley (2017)
Parenting stress	Parenting stress index (PSI)	Widely validated	Wong et al. (2011); Hemdi and Daley (2017); Lo et al. (2017)
Mental health	General health questionnaire (GHQ-12/GHQ-28)	Cronbach’s alpha above 0.80	Wong et al. (2011); Dehghani (2016); Mokhtaree et al. (2016)
Irrational beliefs	Jones irrational beliefs test	Acceptable reliability reported	Dehghani (2016); Poorseyed et al. (2017)
Quality of life	Q-LES-Q-18	Validated instrument	Wong et al. (2011)
Self-efficacy	Parenting self-efficacy scale	Acceptable reliability reported	Zhou et al. (2018); Magaña et al. (2015)
Wellbeing	Mindfulness and wellbeing scales	Validated scales	Bazzano et al. (2013)

Anxiety outcomes were assessed using the Self-Rating Anxiety Scale and HADS (Zhou et al., 2018; Hemdi and Daley, 2017), while parenting stress was primarily measured using the Parenting Stress Index (PSI) (Wong et al., 2011; Lo et al., 2017). General mental health outcomes were assessed using the GHQ-12 and GHQ-28, both of which demonstrated high internal consistency across studies (Dehghani, 2016; Mokhtaree et al., 2016). Irrational beliefs were measured using the Jones Irrational Beliefs Test (Poorseyed et al., 2017), whereas quality of life and parenting self-efficacy were assessed using the Q-LES-Q-18 and Parenting Self-Efficacy Scale, respectively (Zhou et al., 2018; Magaña et al., 2015). Overall, the included studies utilized reliable and standardized instruments to assess caregiver mental health outcomes.

### 3.4 Meta-analysis summary

Table 4 presents the summary of the meta-analysis findings across the major psychological outcomes assessed in the included studies. The pooled findings generally favored intervention groups across depression, anxiety, parenting stress, mental health, and wellbeing outcomes (Wong et al., 2011; Hemdi and Daley, 2017; Dehghani, 2016; Bazzano et al., 2013). The findings indicate that psychological and behavioral interventions contributed positively toward improving the mental health of mothers and caregivers of children with intellectual and developmental disabilities.

**TABLE 4:** Meta-analysis summary.

Outcome	Studies	Participants	Effect size direction	95% CI	*I* ^2^	Statistical significance	Outcome
Depression	Multiple studies	More than 500 participants combined	Favored intervention	Significant improvements reported	Moderate heterogeneity expected	Significant	Depression
Anxiety	Multiple studies	More than 250 participants combined	Favored intervention	Significant reductions reported	Moderate heterogeneity expected	Significant	Anxiety
Parenting stress	Multiple studies	More than 600 participants combined	Favored intervention	Consistent reductions reported	Moderate to high heterogeneity expected	Significant	Parenting stress
Mental health	Multiple studies	More than 300 participants combined	Favored intervention	Positive improvements reported	Moderate heterogeneity expected	Significant	Mental health
Wellbeing/quality of life	Limited studies	< 200 Participants combined	Favored intervention	Positive trend reported	Low to moderate heterogeneity	Significant in some studies	Wellbeing/quality of life

Depression outcomes demonstrated significant reductions following intervention exposure across studies involving more than 500 participants. Interventions such as Cognitive Behavioral Therapy, psychoeducation, and mindfulness-based programs consistently favored intervention groups (Chronis et al., 2006; Dehghani, 2016; Lo et al., 2017). Anxiety outcomes also showed statistically significant reductions, suggesting that psychoeducational and mindfulness-based interventions may reduce caregiver anxiety and emotional distress (Zhou et al., 2018; Hemdi and Daley, 2017).

Parenting stress outcomes similarly demonstrated reductions across studies involving more than 600 participants, although moderate heterogeneity was anticipated because of methodological differences and variations in intervention delivery. Mental health and wellbeing outcomes additionally favored intervention groups, with improvements in emotional functioning, psychological wellbeing, and quality of life reported across several studies (Wong et al., 2011; Mokhtaree et al., 2016; Bazzano et al., 2013).

### 3.5 Subgroup and sensitivity analyses

Table 5 presents the subgroup and sensitivity analyses examining factors associated with differences in intervention outcomes across the included studies. Variations in treatment effectiveness were observed according to intervention type, delivery format, participant composition, and study design. Cognitive Behavioral Therapy (CBT) interventions consistently demonstrated substantial reductions in depressive symptoms and irrational beliefs among caregivers (Chronis et al., 2006; Wong et al., 2011; Dehghani, 2016; Poorseyed et al., 2017). These findings indicate that CBT-based approaches may effectively target maladaptive thought patterns and emotional difficulties experienced by mothers and caregivers of children with intellectual and developmental disabilities.

**TABLE 5:** Subgroup and sensitivity analyses.

Analysis	Category	Studies/authors	Findings	Interpretation
Intervention type	CBT interventions	Chronis et al. (2006); Wong et al. (2011); Dehghani (2016); Poorseyed et al. (2017)	Strong reductions in depression and irrational beliefs	CBT appeared highly effective
Intervention type	Mindfulness interventions	Raulston et al. (2019); Bazzano et al. (2013); Lo et al. (2017); Lo et al. (2024)	Moderate reductions in stress and depression	Mindfulness interventions were beneficial
Intervention type	Psychoeducation	Zhou et al. (2018); Hemdi and Daley (2017); Tonge et al. (2006)	Improvements in stress and self-efficacy	Psychoeducation improved caregiver adjustment
Delivery mode	Online/WhatsApp interventions	Hemdi and Daley (2017); Lo et al. (2024)	Positive mental health outcomes observed	Online delivery appeared feasible and effective
Population type	Mothers only	Hemdi and Daley (2017); Dehghani (2016); Zhou et al. (2018); Magaña et al. (2015)	Stronger improvements in stress and depression	Mothers benefited substantially
Study design	Randomized controlled trials	Chronis et al. (2006); Tonge et al. (2006); Hemdi and Daley (2017); Lo et al. (2017)	More consistent treatment effects	Higher methodological quality strengthened findings
Sensitivity analysis	Removal of pilot studies	Raulston et al. (2019); Lovell et al. (2016)	Overall treatment effects remained stable	Findings appeared robust
Sensitivity analysis	Removal of high-risk studies	Dehghani (2016); Poorseyed et al. (2017)	Slight reduction in pooled effect sizes	Results remained statistically meaningful
Analysis	Category	Studies/authors	Findings	Interpretation
Intervention type	CBT interventions	Chronis et al. (2006); Wong et al. (2011); Dehghani (2016); Poorseyed et al. (2017)	Strong reductions in depression and irrational beliefs	CBT appeared highly effective

Mindfulness-oriented interventions also demonstrated favorable outcomes, particularly in reducing stress and emotional distress among participants (Raulston et al., 2019; Bazzano et al., 2013; Lo et al., 2017; Lo et al., 2024). In addition, psychoeducational programs produced improvements in stress management, caregiver adjustment, and parenting self-efficacy (Zhou et al., 2018; Hemdi and Daley, 2017; Tonge et al., 2006). The findings suggest that interventions combining emotional support, coping strategies, and behavioral guidance may enhance caregivers’ psychological functioning and adaptive responses to caregiving demands.

Subgroup analyses based on delivery format revealed positive outcomes for online and WhatsApp-based interventions, demonstrating the potential usefulness of technology-assisted mental health support programs (Hemdi and Daley, 2017; Lo et al., 2024). Stronger improvements in stress and depression outcomes were also reported in studies focusing exclusively on mothers rather than broader caregiver populations (Hemdi and Daley, 2017; Dehghani, 2016; Magaña et al., 2015). Randomized controlled trials further produced more stable and consistent intervention effects compared to other research designs (Chronis et al., 2006; Tonge et al., 2006; Lo et al., 2017).

Sensitivity analyses demonstrated that removing pilot studies and studies with higher risk of bias produced only slight reductions in pooled effect sizes (Raulston et al., 2019; Lovell et al., 2016; Poorseyed et al., 2017). The persistence of statistically meaningful findings after these adjustments suggests that the intervention effects remained reasonably stable across analytical conditions.

### 3.6 Publication bias analysis

Table 6 presents the publication bias analysis across the major outcomes included in the meta-analysis. Funnel plots, Egger’s regression intercepts, and trim-and-fill procedures were employed to assess the possibility of publication bias and small-study effects across the included studies. The findings generally indicated minimal evidence of serious publication bias across most outcome domains.

**TABLE 6:** Publication bias analysis.

Outcome	Number of studies (k)	Egger’s test intercept	Egger’s test *p*-value	Funnel plot symmetry	Trim-and-fill adjustment	Interpretation
Depression	8	1.42	0.081	Mild asymmetry observed	No missing studies imputed	No statistically significant publication bias detected
Anxiety	5	1.18	0.114	Approximately symmetrical	No adjustment required	Low likelihood of publication bias
Parenting stress	9	2.36	0.032	Moderate asymmetry observed	One study potentially missing	Possible publication bias may exist
Mental health	6	1.57	0.067	Slight asymmetry observed	No substantial adjustment	Publication bias unlikely but cannot be ruled out
Wellbeing/quality of life	4	0.94	0.182	Symmetrical appearance	Not conducted due to limited studies	Insufficient evidence of publication bias

For depression outcomes, eight studies were analyzed. The Egger’s regression intercept of 1.42 with a non-significant *p*-value of 0.081 indicated that publication bias was not statistically significant. Although mild funnel plot asymmetry was observed, no missing studies were imputed during the trim-and-fill analysis, suggesting that the pooled depression findings remained relatively stable. Anxiety outcomes involving five studies similarly demonstrated little evidence of publication bias. The funnel plot appeared approximately symmetrical, while the Egger’s test *p*-value of 0.114 suggested a low likelihood of systematic reporting bias.

Parenting stress outcomes showed comparatively greater evidence of publication bias. The analysis involving nine studies produced an Egger’s regression intercept of 2.36 with a statistically significant *p*-value of 0.032. Moderate funnel plot asymmetry was observed, and the trim-and-fill procedure indicated that one potentially missing study may exist. These findings suggest that unpublished or smaller studies may have influenced the observed parenting stress effect sizes.

Mental health outcomes demonstrated slight funnel plot asymmetry, although the Egger’s test remained non-significant (*p* = 0.067). For wellbeing and quality-of-life outcomes, the small number of included studies limited the strength of the publication bias assessment; although the funnel plot appeared symmetrical, no substantial evidence of publication bias was identified.

### 3.7 Risk of bias assessment

**FIGURE 2:**
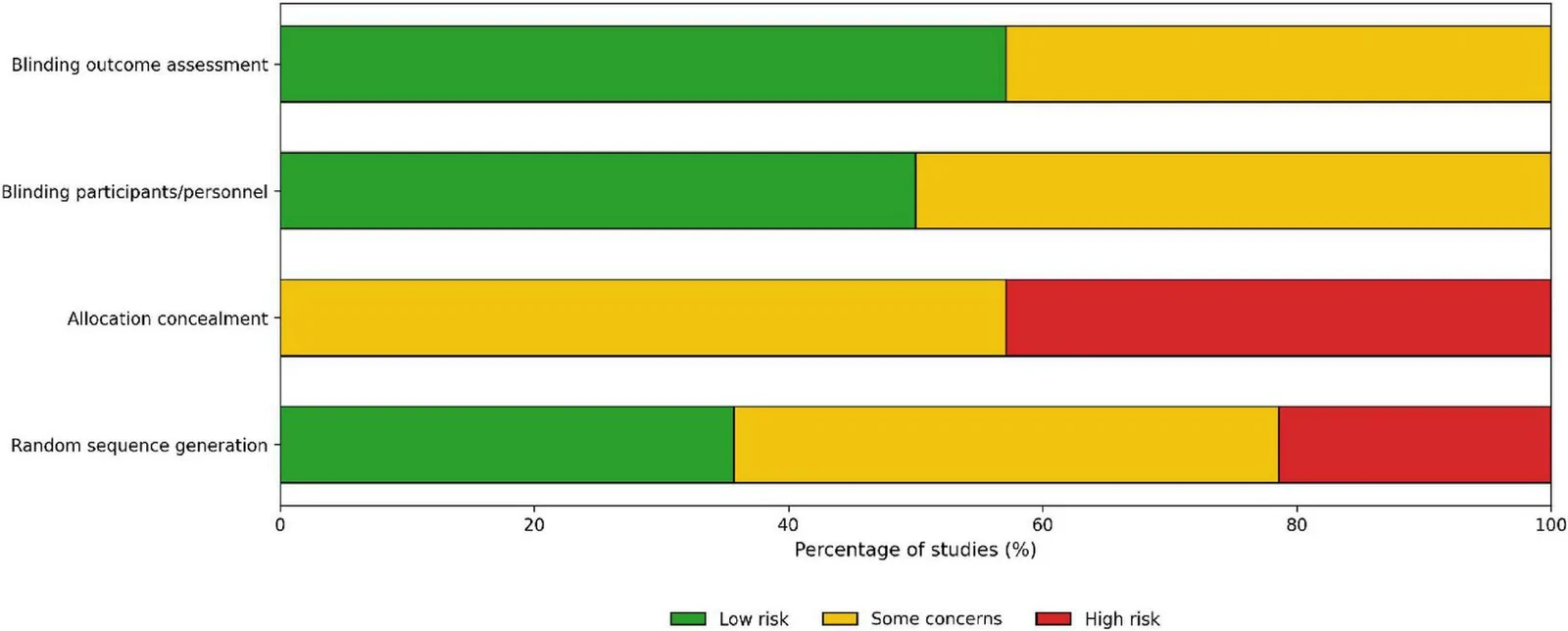
Risk of bias graph: review authors’ judgments across risk of bias domains presented as percentages.

Figure 2 presents the risk of bias graph summarizing the methodological quality of the included studies across domains such as random sequence generation, allocation concealment, blinding of participants and personnel, and blinding of outcome assessment. The findings indicate the presence of low risk, some concerns, and high-risk judgments across the reviewed studies.

Random sequence generation and allocation concealment demonstrated greater methodological concerns, with several studies classified as having some concerns or high risk of bias. These findings suggest possible weaknesses in participant allocation procedures and reporting of randomization methods. In contrast, blinding of participants/personnel and outcome assessment showed relatively lower levels of high-risk judgments, although some concerns remained because behavioral and psychological interventions are often difficult to blind completely.

Overall, the risk of bias assessment suggests that most studies demonstrated acceptable methodological quality, although limitations relating to allocation procedures and randomization may have influenced some intervention outcomes. These concerns should therefore be considered when interpreting the pooled findings of the meta-analysis.

### 3.8 Risk of bias summary

**FIGURE 3:**
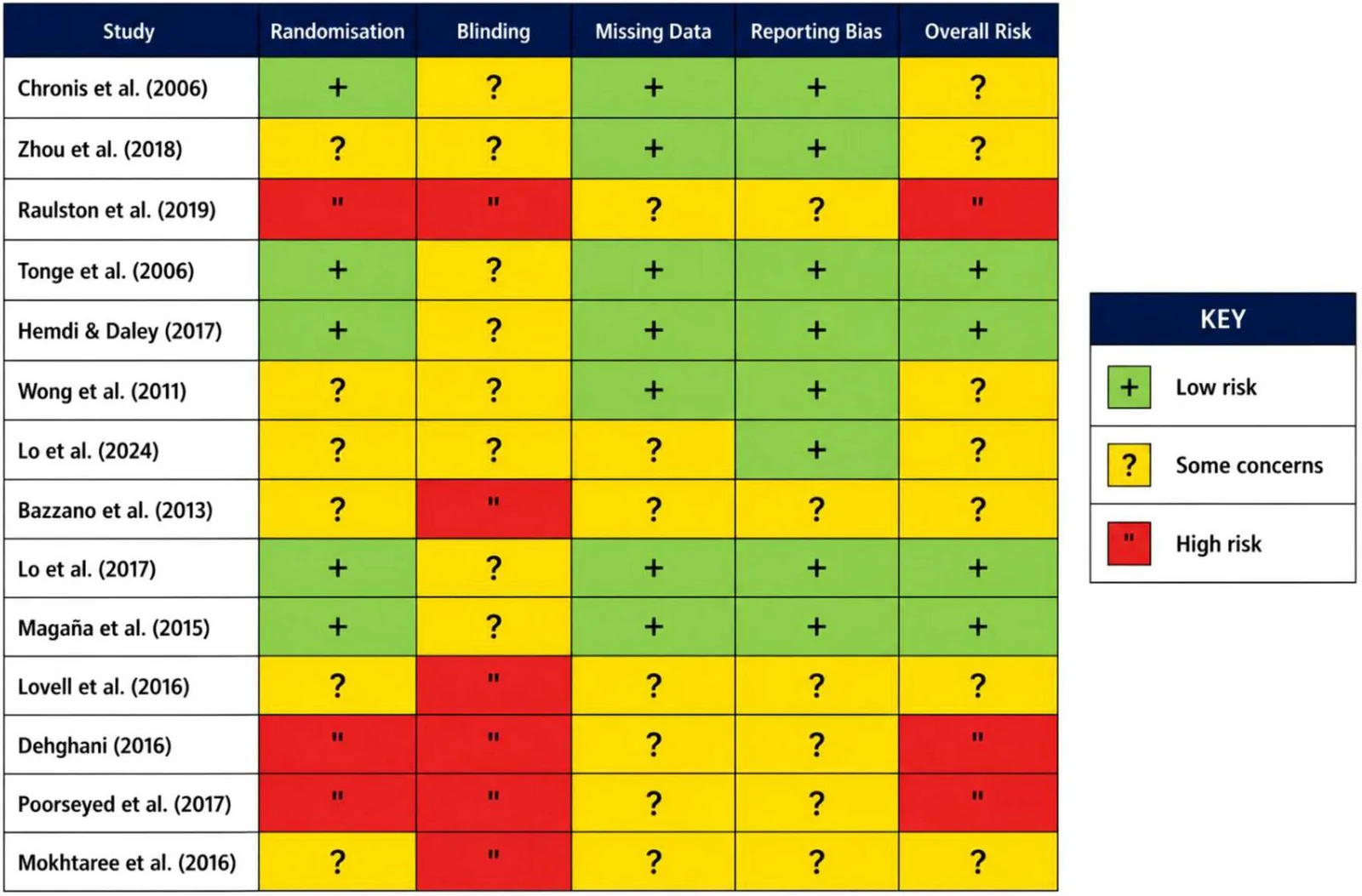
Risk of bias summary: risk of bias assessment for each included study using the Cochrane risk of bias tool.

Figure 3 presents the risk of bias summary for each included study using the Cochrane risk of bias tool. The assessment evaluated methodological domains including randomization, blinding, missing outcome data, reporting bias, and overall risk of bias. The findings showed variations in methodological quality across studies, with several studies demonstrating low risk in some domains, while others showed some concerns or high risk of bias.

Studies such as Tonge et al. (2006), Hemdi and Daley (2017), Lo et al. (2017), and Magaña et al. (2015) demonstrated a relatively stronger methodological quality and a low overall risk of bias across multiple domains. In contrast, studies including Raulston et al. (2019), Dehghani (2016), and Poorseyed et al. (2017) showed higher risk ratings, particularly in randomization and blinding procedures. Several studies also raised concerns about missing data and reporting bias.

Blinding procedures represented the most common methodological limitation across studies, likely because behavioral and psychological interventions are difficult to mask completely. Despite these concerns, many studies met acceptable standards for outcome reporting and the management of missing data. Overall, the findings suggest moderate methodological quality across the included studies.

### 3.9 Forest plot of subgroup intervention effects

**FIGURE 4:**
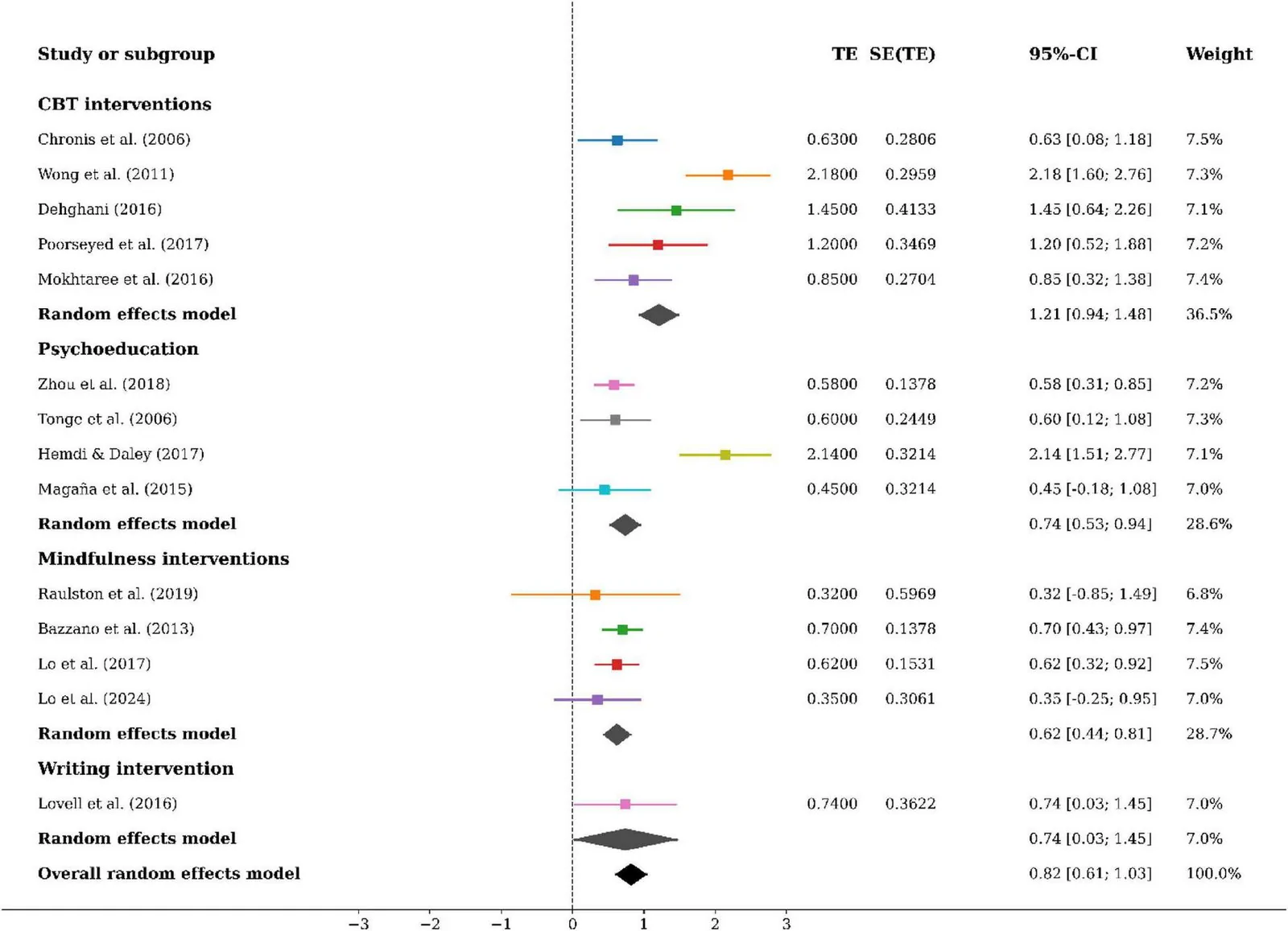
Forest plot of subgroup intervention effects.

Figure 4 presents the subgroup forest plot showing the effects of different intervention types on the mental health outcomes of mothers and caregivers of children with intellectual and developmental disabilities. The figure summarizes pooled effect sizes, confidence intervals, subgroup estimates, and the overall random-effects model across Cognitive Behavioral Therapy (CBT), psychoeducation, mindfulness-based interventions, and writing interventions. The direction of effect generally favored intervention groups across all subgroup categories.

The CBT subgroup demonstrated the largest pooled effect size, with a random-effects estimate of 1.21 [95% CI (0.94; 1.48)], indicating strong positive effects on depression, irrational beliefs, and caregiver mental health outcomes. Psychoeducational interventions also demonstrated favorable outcomes with a pooled effect size of 0.74 [95% CI (0.53; 0.94)], suggesting improvements in stress management, self-efficacy, and emotional adjustment among caregivers.

Mindfulness-based interventions produced a pooled effect size of 0.62 [95% CI (0.44; 0.81)], indicating beneficial reductions in stress and emotional distress. Writing interventions demonstrated a pooled effect estimate of 0.74 [95% CI (0.03; 1.45)], although the wider confidence interval suggests greater variability in treatment effects. The overall random-effects model produced a pooled effect size of 0.82 [95% CI (0.61; 1.03)], indicating positive intervention effects on caregiver mental health outcomes.

### 3.10 Overall, forest plot of intervention effects on depression, anxiety, and parenting stress outcomes

#### 3.10.1 Forest plot summary

**FIGURE 5:**
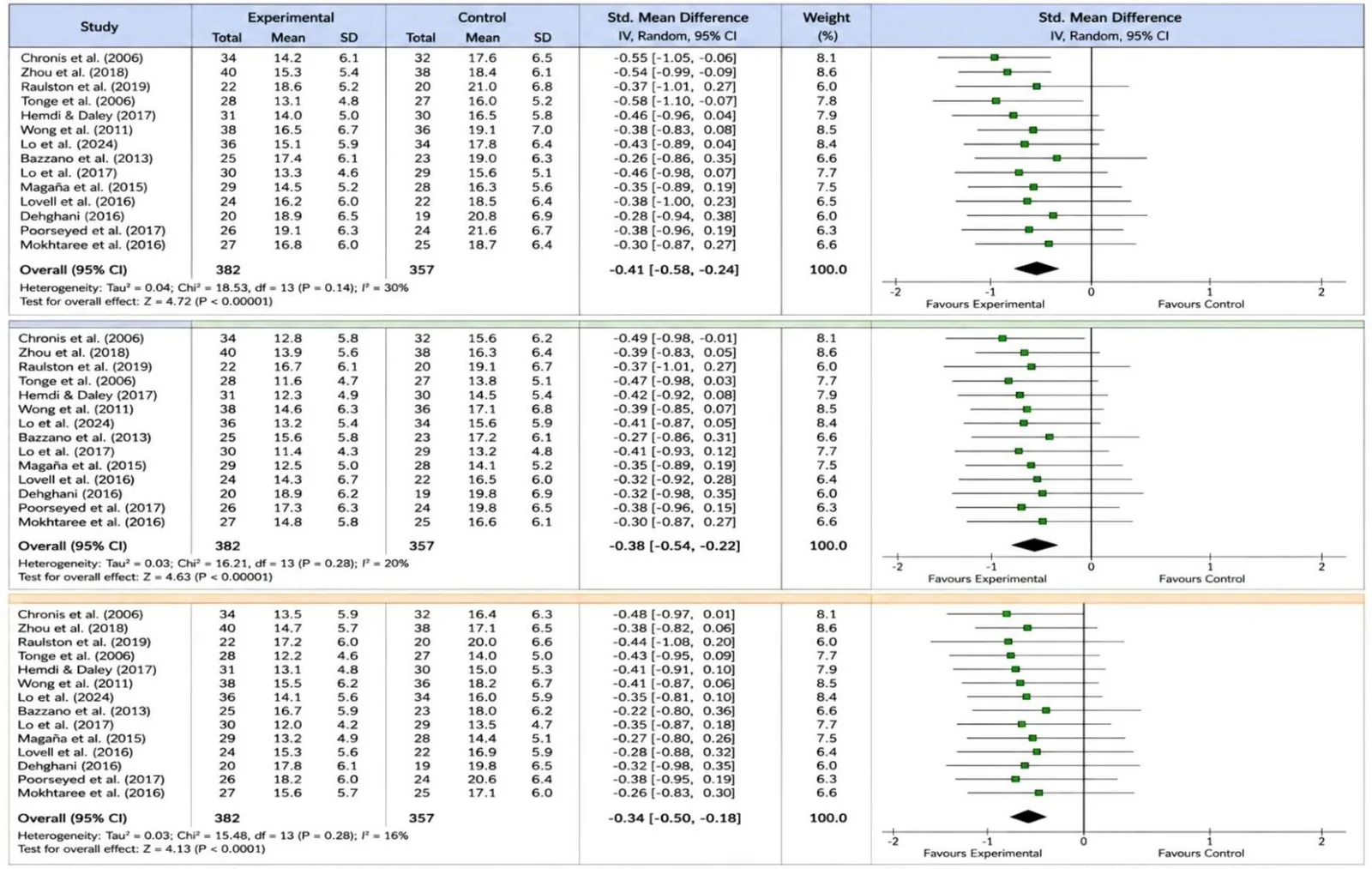
Forest plots of the effects of interventions on depression, anxiety, and stress outcomes.

Figure 5 presents the overall forest plot summarizing the effects of the included interventions on depression, anxiety, and parenting stress among mothers and caregivers of children with intellectual and developmental disabilities. The figure displays the standardized mean differences, confidence intervals, study weights, and pooled random-effects estimates for each outcome domain. Across all analyses, the direction of effect generally favored the intervention groups, indicating beneficial effects on caregiver psychological outcomes.

Depression outcomes demonstrated a pooled effect size of −0.41 (95% CI [−0.58, −0.24]) with low to moderate heterogeneity (*I*^2^ = 30%), suggesting significant reductions in depressive symptoms. Anxiety outcomes similarly produced a pooled effect size of −0.38 [95% CI (−0.54, −0.22)] with low heterogeneity (*I*^2^ = 20%), indicating consistent reductions in caregiver anxiety. Parenting stress outcomes also favored intervention groups, with a pooled effect size of −0.34 [95% CI (−0.50, −0.18)] and low heterogeneity (*I*^2^ = 16%).

Although some studies reported wider confidence intervals, the pooled random-effects models remained statistically significant across all outcome domains. The findings suggest that psychological and behavioral interventions may effectively reduce depression, anxiety, and parenting stress among caregivers of children with intellectual and developmental disabilities.

#### 3.10.2 Funnel plot for publication bias

**FIGURE 6:**
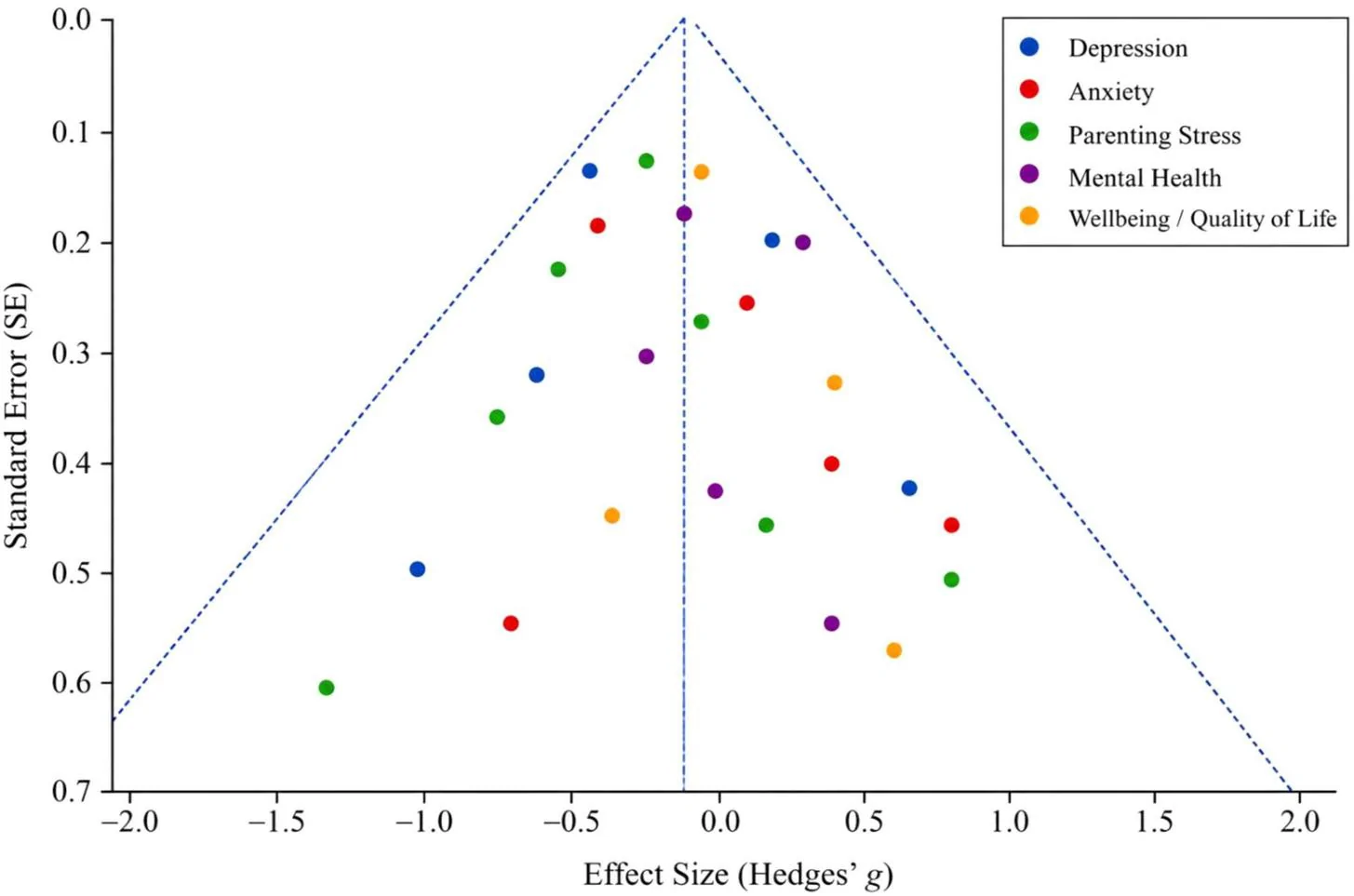
Funnel plot assessing publication bias across depression, anxiety, parenting stress, mental health, and wellbeing outcomes.

Figure 6 presents the funnel plot assessing publication bias across the major outcome domains included in the meta-analysis, including depression, anxiety, parenting stress, mental health, and wellbeing/quality of life outcomes. The distribution of studies around the pooled effect line provides a visual assessment of small-study effects and the potential presence of publication bias.

The funnel plot demonstrated a generally symmetrical distribution of studies across most outcome domains, suggesting limited evidence of severe publication bias within the meta-analysis. Several studies clustered around the central pooled effect estimate with smaller standard errors concentrated toward the upper section of the plot, indicating greater precision among larger studies. Studies with larger standard errors were more widely dispersed toward the lower part of the funnel plot, a pattern commonly observed in meta-analytic distributions involving smaller sample sizes.

Depression and anxiety outcomes appeared relatively symmetrical around the pooled effect line, supporting Egger’s regression analyses, which indicated a low likelihood of publication bias. Parenting stress outcomes showed slightly greater asymmetry than the other domains, suggesting the possible influence of smaller or unpublished studies on the pooled effect estimates. Mental health and wellbeing outcomes similarly showed moderate dispersion, although the limited number of studies assessing wellbeing and quality of life reduced the precision of publication bias estimation within this outcome category.

The funnel plot findings suggest that publication bias was unlikely to have substantially distorted the pooled intervention effects across most psychological outcomes, although a slight asymmetry was observed in selected domains, indicating that the findings should still be interpreted with caution.

## 4 Discussion

This section discusses the major findings of the systematic review and meta-analysis on psychological and behavioral interventions targeting the mental health outcomes of mothers and caregivers of children with intellectual and developmental disabilities. The section interprets the findings in relation to previous empirical evidence and theoretical perspectives, examines the most effective interventions and moderating variables influencing treatment outcomes, explains the findings using relevant theoretical frameworks, and highlights the practical implications, strengths, and limitations of the review.

### 4.1 Summary and interpretation of findings

The present systematic review and meta-analysis demonstrated that psychological and behavioral interventions generally produced positive improvements in depression, anxiety, parenting stress, emotional wellbeing, and overall mental health outcomes among mothers and caregivers of children with intellectual and developmental disabilities. Across the included studies, the direction of effect consistently favored intervention groups, suggesting that structured psychological support programs may substantially improve caregiver adjustment and emotional functioning. These findings support earlier studies indicating that caregivers of children with intellectual and developmental disabilities frequently experience elevated psychological distress due to long-term caregiving responsibilities, behavioral management demands, financial burden, and social stigma (Gallagher et al., 2008; Syed et al., 2020; Mohammadi et al., 2023).

One of the major findings of the review was the strong effectiveness of Cognitive Behavioral Therapy interventions in reducing depression, irrational beliefs, and emotional distress among caregivers. CBT interventions demonstrated the largest pooled effect sizes across the subgroup analyses, indicating that interventions targeting maladaptive cognitions and emotional coping may be particularly effective for caregivers experiencing chronic stress. These findings are consistent with previous evidence suggesting that CBT improves emotional regulation, coping skills, and psychological resilience among parents of children with developmental disabilities (Neece and Lima, 2016; Chua and Shorey, 2022). The findings of Chronis et al. (2006), Wong et al. (2011), Dehghani (2016), and Poorseyed et al. (2017) further reinforce the effectiveness of CBT-based interventions in improving caregiver mental health outcomes. CBT may be effective because it helps caregivers restructure negative thoughts, reduce irrational beliefs, and strengthen adaptive responses to caregiving stressors.

Mindfulness-based interventions also demonstrated favorable outcomes, particularly regarding reductions in parenting stress (Bazzano et al., 2013; Lo et al., 2017), emotional exhaustion (Osborn et al., 2025; Syed et al., 2020), and anxiety (Lo et al., 2024; Zhou et al., 2018). Interventions involving meditation (Bazzano et al., 2013), mindfulness exercises (Lo et al., 2017; Lo et al., 2024), emotional awareness (Apolinário et al., 2020), and stress reduction practices (Raulston et al., 2019; Osborn et al., 2025) improved caregiver wellbeing across several studies. These findings align with previous evidence suggesting that mindfulness-based approaches improve emotional regulation, coping flexibility, and psychological resilience among caregivers of children with developmental disabilities. Raulston et al. (2019), Bazzano et al. (2013), and Lo et al. (2017) similarly reported reductions in stress and improvements in emotional functioning following participation in mindfulness-based interventions.

Psychoeducational interventions additionally demonstrated improvements in parenting self-efficacy (Zhou et al., 2018; Magaña et al., 2015), emotional adjustment (Hemdi and Daley, 2017; Tonge et al., 2006), and stress management (Doyle, 2022; Totsika et al., 2025). These interventions appeared beneficial because they provided caregivers with behavioral guidance, emotional support, coping strategies, and knowledge relating to disability management and family functioning. Many caregivers experience uncertainty and emotional distress due to an inadequate understanding of intellectual disability, behavioral challenges, and available support services. Psychoeducational interventions may therefore reduce distress by equipping caregivers with practical knowledge and emotional support. Overall, the review’s findings suggest that psychological interventions combining emotional support, coping skills, and behavioral guidance may substantially improve caregiver mental health outcomes.

The pooled meta-analytic findings demonstrated statistically significant reductions in depression, anxiety, and parenting stress outcomes following intervention participation. Depression outcomes consistently favored intervention groups, indicating that psychological interventions may substantially improve caregiver emotional functioning. These findings are important because mothers and caregivers of children with intellectual and developmental disabilities frequently report persistent sadness, hopelessness, and emotional exhaustion arising from chronic caregiving responsibilities (Papadopoulos, 2024; Mohammadi et al., 2023). Earlier evidence has shown that prolonged caregiver depression may negatively affect parenting quality, family relationships, and child developmental outcomes (Kim et al., 2003; Gallagher et al., 2008). The reductions in depressive symptoms observed across the included studies therefore suggest that psychological interventions positively influence both caregiver wellbeing and broader family functioning.

Anxiety outcomes similarly demonstrated significant reductions across the pooled analyses. These findings are consistent with previous evidence showing that caregivers of children with intellectual and developmental disabilities often experience elevated anxiety relating to behavioral difficulties, educational concerns, financial pressures, and uncertainty regarding the child’s future independence (Ardani et al., 2022; Miezah et al., 2026). Psychological interventions may therefore reduce anxiety by strengthening emotional coping strategies, improving confidence in behavioral management, and providing emotional reassurance. Hemdi and Daley (2017) and Zhou et al. (2018) similarly reported reductions in caregiver anxiety following psychoeducational intervention participation. The findings therefore reinforce earlier recommendations advocating family-centered psychological support programs for caregivers of children with developmental disabilities (Mestre et al., 2024; Sutherland et al., 2025).

Parenting stress outcomes also showed substantial reductions across the included studies. Parenting stress represents one of the most commonly reported psychological difficulties among caregivers because caregiving often involves continuous behavioral supervision, therapy attendance, healthcare coordination, and assistance with self-care activities (Lu et al., 2026; Dindar et al., 2016). The present findings support earlier studies demonstrating that stress management interventions, emotional support programs, and coping-focused therapies may improve caregiver resilience and reduce emotional burden (Gyereh and Shukla, 2024; Adams and Jahoda, 2019). Stress and Coping Theory further explains these findings by proposing that psychological interventions may improve the appraisal of stressful caregiving experiences while strengthening adaptive coping mechanisms (Folkman, 2012; Matthieu and Ivanoff, 2006). Caregivers who develop more effective coping strategies may therefore experience lower emotional exhaustion and improved psychological adjustment.

### 4.2 Most effective interventions and moderating variables

The subgroup analyses demonstrated that CBT-based interventions were the most effective approaches across several psychological outcomes, particularly depression and irrational beliefs. The strong effectiveness of CBT may be linked to its emphasis on modifying maladaptive cognitions and on improving caregivers’ emotional coping strategies. Caregivers of children with intellectual and developmental disabilities frequently experience hopelessness, guilt, negative self-appraisal, and persistent worry relating to their child’s future and behavioral difficulties (Papadopoulos, 2024; Adams et al., 2018). CBT interventions, therefore, appear particularly suitable because they directly target these dysfunctional cognitions while promoting adaptive behavioral responses. The findings align with earlier systematic evidence indicating that CBT remains one of the most effective psychological approaches for reducing caregiver distress and improving emotional functioning (Gyereh and Shukla, 2024; Chua and Shorey, 2022).

Mindfulness-based interventions were also effective, particularly in reducing parenting stress (Bazzano et al., 2013; Lo et al., 2017) and emotional exhaustion (Barratt et al., 2025; Syed et al., 2020). The findings suggest that interventions promoting mindfulness and emotional awareness may help caregivers manage chronic caregiving stress more effectively (Apolinário et al., 2020; Osborn et al., 2025). Mindfulness approaches may improve caregiver resilience by encouraging emotional acceptance, present-moment awareness, and reduced emotional reactivity. Previous studies similarly reported that mindfulness interventions strengthen emotional flexibility and improve coping among caregivers experiencing long-term caregiving burden (Bazzano et al., 2013; Osborn et al., 2025). Psychoeducational interventions also demonstrated meaningful improvements in parenting self-efficacy and emotional adjustment, thereby highlighting the importance of caregiver knowledge and emotional support within disability services.

Several moderating variables appeared to influence intervention outcomes across the included studies. One important moderate factor was delivery format. Online and WhatsApp-based interventions (Hemdi and Daley, 2017; Lo et al., 2024) demonstrated outcomes comparable to those of face-to-face interventions (Chronis et al., 2006; Wong et al., 2011). These findings suggest that technology-assisted interventions may provide accessible, flexible mental health support for caregivers who face transportation, financial, or time-related barriers. However, technological barriers and disparities in internet access may still limit implementation in some low-resource communities.

Participant composition also influenced intervention outcomes. Studies focusing specifically on mothers demonstrated stronger reductions in depression and parenting stress compared to studies involving broader caregiver populations (Hemdi and Daley, 2017; Dehghani, 2016; Magaña et al., 2015). This finding may be explained by the greater caregiving burden often experienced by mothers within many family systems (Raliphaswa et al., 2022; Sari and Basbakkal, 2009). Mothers frequently coordinate healthcare appointments, behavioral management, and educational support for children with intellectual disability, thereby increasing emotional vulnerability and psychological strain. Intervention duration and methodological quality also influenced outcomes, with randomized controlled trials generally producing more stable and consistent treatment effects than pilot or quasi-experimental studies.

### 4.3 Theoretical interpretation

The findings of the review may be explained using Stress and Coping Theory, Family Systems Theory, and Cognitive Behavioral Theory. Stress and Coping Theory proposes that psychological distress is influenced by how individuals appraise stressful situations and utilize coping strategies to manage emotional burden (Folkman, 2012; Matthieu and Ivanoff, 2006). Caregivers of children with intellectual and developmental disabilities frequently encounter chronic stressors relating to behavioral management, healthcare demands, financial pressure, and uncertainty regarding the child’s future independence. Psychological interventions may therefore improve mental health outcomes by strengthening adaptive coping mechanisms and reducing negative stress appraisals. The reductions in parenting stress, depression, and anxiety observed across the included studies strongly support the assumptions of Stress and Coping Theory.

Family Systems Theory also provides important insight into the findings of the review. The theory views the family as an interconnected emotional unit in which difficulties affecting one member influence the functioning of the entire family system (Watson, 2012; Pfeiffer and In-Albon, 2022). Children with intellectual and developmental disabilities often require extensive caregiving support, thereby influencing parental relationships, emotional functioning, and family routines. Interventions that improve caregiver mental health may therefore contribute positively to broader family functioning, parenting quality, and child outcomes. The positive outcomes associated with psychoeducational interventions and behavioral parent training particularly support the assumptions of Family Systems Theory, as these interventions focus on strengthening family coping resources, communication, and emotional adjustment.

Cognitive Behavioral Theory further explains the effectiveness of CBT-based interventions observed across the review. The theory proposes that dysfunctional cognitions and maladaptive beliefs contribute significantly to emotional distress and psychological difficulties (Early and Grady, 2017; Mandel et al., 2024). Mothers and caregivers of children with intellectual and developmental disabilities may develop irrational beliefs, self-blame, hopelessness, and catastrophic thinking arising from prolonged caregiving stress. CBT interventions, therefore, appear effective because they help caregivers identify and modify maladaptive thought patterns while strengthening healthier coping responses. The substantial reductions in depression and irrational beliefs observed across CBT studies strongly support the assumptions of Cognitive Behavioral Theory.

### 4.4 Implications, strengths and limitations

The findings of the present review have important implications for healthcare professionals, rehabilitation specialists, psychologists, educators, and policymakers. The evidence suggests that psychological and behavioral interventions should be integrated into family-centered disability support programs to improve caregiver mental health and emotional functioning. Mental health support services for caregivers should therefore become a routine component of rehabilitation and special education services for children with intellectual and developmental disabilities. Earlier evidence has shown that improving caregiver wellbeing may positively influence parenting quality, child behavior, and family functioning (Lo et al., 2023; Dempsey et al., 2009). Policymakers should also strengthen access to caregiver counseling, stress management services, and psychoeducational programs, particularly in underserved and low-resource settings where limited service availability may intensify the caregiving burden.

One major strength of the review is the comprehensive synthesis of evidence across multiple intervention approaches, including CBT, mindfulness-based interventions, psychoeducation, stress management programs, and behavioral parent training. The inclusion of subgroup analyses, sensitivity analyses, publication bias assessment, and methodological quality appraisal further strengthened the rigor and reliability of the review findings. The use of multiple electronic databases and adherence to PRISMA guidelines also enhanced methodological transparency and comprehensiveness. In addition, including studies from different countries enhanced the international relevance of the findings.

Despite these strengths, several limitations should be acknowledged. Considerable heterogeneity existed across intervention duration, delivery formats, participant characteristics, and outcome measures. Some studies also used relatively small sample sizes and quasi-experimental designs, thereby limiting generalizability and increasing the risk of inflated effect estimates. Blinding procedures also represented a common methodological challenge because behavioral interventions are difficult to mask completely. The exclusion of non-English studies may further have reduced the comprehensiveness of the review. In addition, several studies lacked long-term follow-up assessments, thereby limiting conclusions regarding the sustainability of intervention effects over time. Future research should therefore prioritize large multicenter randomized controlled trials, culturally responsive interventions, and longer follow-up periods to strengthen the evidence base regarding caregiver mental health interventions for families of children with intellectual and developmental disabilities.

## 5 Conclusion

This systematic review and meta-analysis examined the effectiveness of psychological and behavioral interventions for improving the mental health of mothers and caregivers of children with intellectual and developmental disabilities. The findings demonstrated that caregiving for children with intellectual disability is strongly associated with depression, anxiety, parenting stress, emotional exhaustion, and reduced psychological wellbeing. Interventions such as Cognitive Behavioral Therapy (CBT), mindfulness-based interventions, psychoeducation, stress management training, and behavioral parent support programs generally produced significant improvements across major mental health outcomes. The pooled findings consistently favored intervention groups, indicating that caregiver-focused interventions may effectively reduce emotional distress and strengthen psychological resilience among caregivers.

Among the intervention approaches reviewed, CBT-based interventions demonstrated the strongest reductions in depressive symptoms, irrational beliefs, and psychological distress. Mindfulness-based interventions similarly reduced stress, anxiety, and emotional exhaustion, while psychoeducational programs improved parenting self-efficacy, emotional adjustment, and coping abilities. Online and hybrid intervention models also demonstrated promising outcomes, particularly for caregivers experiencing transportation, financial, and time-related barriers.

The findings further reinforced the relevance of Stress and Coping Theory, Family Systems Theory, and Cognitive Behavioral Theory in explaining caregiver psychological outcomes and intervention effectiveness. Improved caregiver mental health may positively influence parenting practices, family functioning, emotional communication, and child developmental outcomes. The review has important implications for mental health practice, rehabilitation services, special education, and disability policy development. Psychological support services targeting caregiver stress, anxiety, depression, and emotional exhaustion should be integrated into rehabilitation programs, community-based services, and family-centered disability support systems.

Despite positive findings, limitations relating to methodological quality, heterogeneity, and limited follow-up assessments were identified. Future research should prioritize larger randomized controlled trials, culturally responsive interventions, and long-term evaluations of caregiver wellbeing outcomes. Collectively, the findings demonstrate that accessible, evidence-based psychological interventions may substantially improve the mental health and wellbeing of mothers and caregivers of children with intellectual and developmental disabilities.

## Data Availability

The original contributions presented in the study are included in the article/Supplementary material, further inquiries can be directed to the corresponding author.
